# Transplantation of glutamatergic neuronal precursor cells in the paraventricular thalamus and claustrum facilitates awakening with recovery of consciousness

**DOI:** 10.1111/cns.14137

**Published:** 2023-03-07

**Authors:** Tong Zhao, Naili Wei, Tianwen Li, Kezhu Chen, Wenqiang Cui, Zhifu Wang, Fan Wang, Yuanxiang Lin, Jianhong Zhu

**Affiliations:** ^1^ Department of Neurosurgery, The First Affiliated Hospital, Neurosurgery Research Institute Fujian Medical University Fuzhou Fujian China; ^2^ Department of Neurosurgery, State Key laboratory of Medical Neurobiology, Shanghai Key Laboratory of Brain Function and Regeneration, Shanghai Medical College, Fudan University Huashan Hospital, Institute of Brain Science Fudan University Shanghai China; ^3^ Department of Neurosurgery The First Affiliated Hospital of Shantou University Medical College Shantou Guangdong China; ^4^ Department of Neurology Affiliated Hospital of Shandong University of Traditional Chinese Medicine Jinan Shandong China; ^5^ Department of Neurology Peking University Third Hospital Beijing China

**Keywords:** claustrum, disorders of consciousness, glutamatergic neuronal precursor cells, paraventricular thalamus, stem cell, transplant, traumatic brain injury

## Abstract

**Background:**

Stem cells offer a promising therapeutic strategy for patients with disorders of consciousness (DOC) after severe traumatic brain injury (TBI), but the optimal transplantation sites and cells are not clear. Although the paraventricular thalamus (PVT) and claustrum (CLA) are associated with consciousness and are candidate transplantation targets, few studies have been designed to investigate this possibility.

**Methods:**

Controlled cortical injury (CCI) was performed to establish a mouse model of DOC. CCI‐DOC paradigm was established to investigate the role of excitatory neurons of PVT and CLA in disorders of consciousness. The role of excitatory neuron transplantation in promoting arousal and recovery of consciousness was determined by optogenetics, chemogenetics, electrophysiology, Western blot, RT‐PCR, double immunofluorescence labeling, and neurobehavioral experiments.

**Results:**

After CCI‐DOC, neuronal apoptosis was found to be concentrated in the PVT and CLA. Prolonged awaking latency and cognitive decline were also seen after destruction of the PVT and CLA, suggesting that the PVT and CLA may be key nuclei in DOC. Awaking latency and cognitive performance could be altered by inhibiting or activating excitatory neurons, implying that excitatory neurons may play an important role in DOC. Furthermore, we found that the PVT and CLA function differently, with the PVT mainly involved in arousal maintenance while the CLA plays a role mainly in the generation of conscious content. Finally, we found that by transplanting excitatory neuron precursor cells in the PVT and CLA, respectively, we could facilitate awakening with recovery of consciousness, which was mainly manifested by shortened awaking latency, reduced duration of loss of consciousness (LOC), enhanced cognitive ability, enhanced memory, and improved limb sensation.

**Conclusion:**

In this study, we found that the deterioration in the level and content of consciousness after TBI was associated with a large reduction in glutamatergic neurons within the PVT and CLA. Transplantation of glutamatergic neuronal precursor cells could play a beneficial role in promoting arousal and recovery of consciousness. Thus, these findings have the potential to provide a favorable basis for promoting awakening and recovery in patients with DOC.

## Graphical abstract

The deterioration in the level and content of consciousness after TBI was associated with a large reduction in glutamatergic neurons within the PVT and CLA. Transplantation of glutamatergic neuronal precursor cells could play a beneficial role in promoting arousal and recovery of consciousness. These findings have the potential to provide a favorable basis for promoting awakening and recovery in patients with DOC.

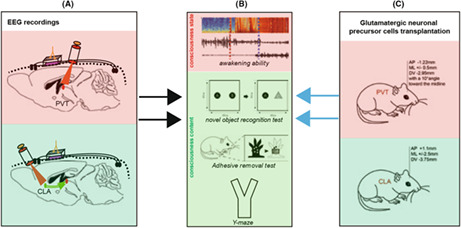

## 1 INTRODUCTION

Disorders of consciousness (DOC) are a common complication after brain injury. Some patients with DOC do not recover their consciousness; instead, they remain in a vegetative state. Such patients are completely incapacitated and require continuous professional medical care.^1^ The average survival time of patients with DOC is only 2–5 years.^2^ The incidence of traumatic brain injury (TBI) is as high as 13/100,000; the mortality rate of severe TBI is approximately 50% and 10%–15% of patients with severe TBI develop DOC.^3^, ^4^ Thus, approximately 40,000 people worldwide develop DOC following TBI in a given year. At present, many treatments, including pharmacotherapy, hyperbaric oxygen therapy, and neuromodulation techniques (deep brain electrical stimulation,^5^, ^6^ spinal cord electrical stimulation,^7^, ^8^ and cortical nerve stimulation^9^), are used to treat DOC following TBI, but their efficacy is highly variable. With the development of stem cell technology, stem cells have taken on an important therapeutic role as “seed cells^10^” or “carrier cells^11^, ^12^” in clinical practice. In fact, stem cell therapy has the potential as a treatment for DOC^13^. At present, stem cell transplantation for the treatment of DOC is still in the exploratory stage and faces two main problems: the selection of transplanted cells and the identification of optimal transplantation targets.

In 2018, it was reported that the paraventricular thalamus (PVT) is a key nucleus for maintaining wakefulness and parsing the neural circuit mechanisms involving the PVT^14^; almost at the same time, another study elaborated on the important role of the PVT in encoding information in the brain.^15^ Anatomically, the PVT is located in the midline region of the thalamus, which receives a large number of neural afferents from the hypothalamus and brainstem, has close bidirectional neural projections to the insula and prefrontal lobes, and is the hub of top‐down neural network regulation and information transmission in the brain.^16^ The findings of these studies suggest that the PVT may be a key nucleus in the development of DOC.

Koch suggested that the claustrum is connected bidirectionally with most cortical regions, and the claustrum (CLA) was hypothesized to play a critical part in information integration leading to consciousness.^17^ Koubeissi reported that the stimulation of the CLA by an electrical current reverses the state of consciousness (from awake to comatose and back again) in epileptic patients. The findings from another experiment also support the possible association of the CLA with consciousness.^18^ In that study, scientists examined 171 veterans with traumatic cranial injury and assessed the effects of CLA damage on consciousness; they found that CLA damage was associated with the duration of loss of consciousness (LOC).^19^

In light of the above studies, we hypothesized that there are neural network correlates of consciousness,^20^ in which the PVT is one of the nuclei controlling awakening, a process that is closely related to the maintenance of consciousness; we also hypothesized that the CLA is the key channel of the consciousness circuitry and one of the hubs of connectivity among various parts, controlling the transmission and integration of information. Excitatory neurons are the smallest units involved in the neural network of consciousness. Injury to excitatory neurons might affect the regulatory function of the CLA and PVT, leading to the dysfunction of the consciousness‐related neural network, which eventually leads to DOC.

In this study, we explored the roles of the PVT and CLA in DOC and determined which cells play major roles in these nuclei, thus informing the choice of transplanted cells to treat DOC, clarifying whether the PVT and CLA can be used as targets for cell transplantation and explaining the role of excitatory neurons in the neural network of consciousness.

## 2 MATERIALS AND METHODS

### 2.1 Animals

All animal experiments followed the Guide for the Care and Use of Laboratory Animals (8th edition, revised in 2011) and were approved by the Animal Ethics Committee of Fudan University. C57BL6/J mice were used as the genetic background of our mouse models. Adult male mice aged 6–8 weeks were used in this study.

### 2.2 Cell culture

#### 2.2.1 iPSCs

We obtained iPSCs by introducing four factors (Oct3/4, Sox2, hc‐Myc, and hKlf4) into 9021 human fibroblast cell lines with the CytoTune‐iPS 2.0 Sendai Reprogramming Kit. The MOIs for the four factors were as follows: human Oct3/4 = 5, human Sox2 = 5, hc‐Myc = 5, and hKlf4 = 3.

#### 2.2.2 Glutamatergic neuronal precursor cells

Glutamatergic neurons were induced by inhibiting the SMAD pathway by adding 1 μM dorsomorphin and 10 μM SB431542 to the culture medium of iPSCs.

### 2.3 Establishment of the CCI‐DOC mouse model

**FIGURE 1:**
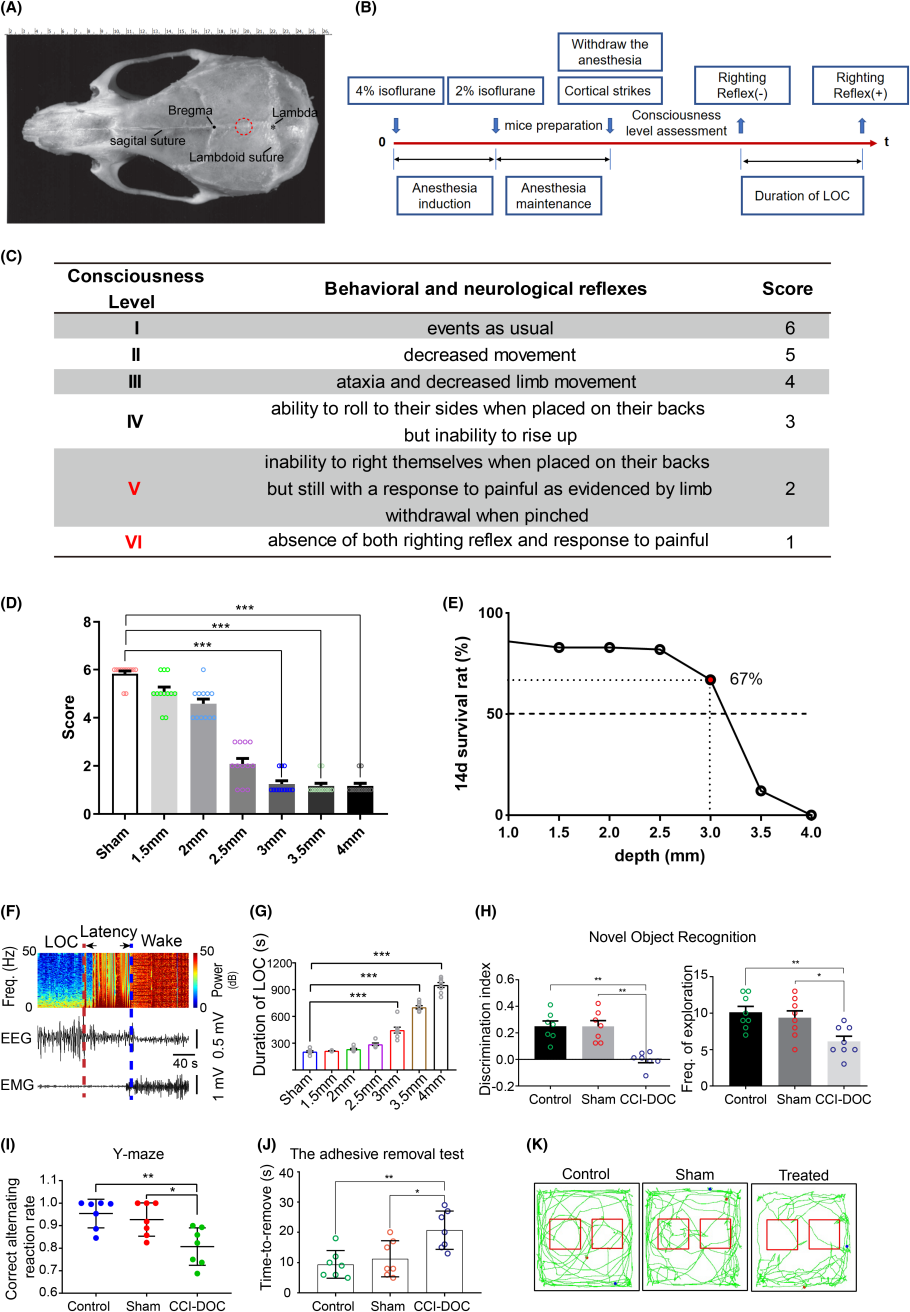
(A) The surface markings of the skulls of mice. The red circle in the picture is the strike position. (B) Flow chart of the CCI‐DOC model. (C) Scoring of different states of consciousness. (D) Consciousness scores at different strike depths (*n* = 12 for each group), ***p < 0.001. (E) Survival rate of experimental animals at 14 days with different strike depths (*n* = 12 for each group). The survival rate of experimental animals in the 3 mm group was 67%. (F) EEG–EMG recording in mice after LOC. (G) Duration of LOC in mice at different strike depths (*n* = 7 for each group). (H) Decreased discrimination index (*n* = 7 for each group) and decrease in the number of explorations of the novel object after LOC (*n* = 7 for each group). (I) Decreased memory capacity (*n* = 7 for each group). (J) Significant reduction in limb sensation (*n* = 7 for each group). (K) Trajectory diagram of the novel object recognition experiment.

Controlled cortical injury (CCI) is a commonly used and highly regarded model of brain trauma that induces reproducible and well‐controlled injury.^21^ Healthy male C57BL/6 mice 6–8 weeks of age were anesthetized with an animal gas anesthesia apparatus at an induction concentration of 4% isoflurane and a maintenance concentration of 2%. The mice were fixed on a controlled cortical impactor (Hatteras PinPoint™ PCI3000) stereotactic device in the prone position. The top of the head was shaved, the area was routinely disinfected with Anl iodine, the scalp was anesthetized with local infiltration of lidocaine, the skin was incised at the midline of the head (the incision was approximately 1 cm), the soft tissue and the outer membrane of the bone were bluntly peeled away, and the skull was exposed. A circular bone window with a diameter of 6 mm was created at the right side of the midpoint between the fontanelle and the herringbone point (Figure 1A). The impingement device was set at 3 mm above the dura, with an impact speed of 3 m/s, and the duration was 180 ms. The anesthetic machine was withdrawn immediately after the impact, and a consciousness assessment was performed (Figure 1B). In the Sham group, the bone flap was removed without cortical impact. All mice were singly housed after the operations, and the temperature was maintained at 25°C. The airway was unobstructed, and aspiration was performed when necessary. An appropriate amount of saline was injected intraperitoneally to reduce mortality.

### 2.4 Consciousness level assessment

The modified consciousness level assessment criteria^22^ (Figure 1D) were as follows:

**Stage I,** events as usual.

**Stage II,** decreased movement.

**Stage III,** ataxia and decreased limb movement.

**Stage IV,** ability to roll to their sides when placed on their backs but inability to rise up.

**Stage V,** inability to right themselves when placed on their backs but still with a response to pain, as evidenced by limb withdrawal when pinched.

**Stage VI,** absence of both righting reflex and response to pain.

Animals were considered unconscious if classified as stage V or VI. The duration of loss of consciousness was the time between the disappearance and the recovery of the righting reflex (Figure 1B). We assigned each level of consciousness a corresponding score for statistical purposes, where levels I, II, III, IV, V, and VI were scored 6, 5, 4, 3, 2, and 1, respectively.

### 2.5 Immunofluorescence staining

The mice were fixed in the supine position after deep anesthesia, and the chest was opened rapidly to fully expose the heart. First, approximately 100 mL of prechilled 4°C physiological saline was perfused, and after the rat's two forelimbs and two lungs turned white, they were perfused with prechilled (4°C) 4% paraformaldehyde solution. The brains were bathed in 4% PFA for 90 min, dehydrated in 30% sucrose, and embedded in optimal cutting temperature (OCT; Sakura) compound. The brains were cut into 20 μm sections. The cryosections were air‐dried for 40 min at room temperature and incubated in PBS with 3% donkey serum and 0.1% Triton X‐100 for 30 min. After incubating with primary antibodies overnight at 4°C, the sections were washed with PBS three times. Cryosections were incubated with secondary antibodies for 30 min at room temperature and washed with PBS. Mounting media (Vector Laboratories) was used for counterstaining. All samples were observed and photographed with a Nikon (A1^+^) confocal microscope.

The following primary antibodies were used: vGlut1 (rabbit, 1:500; PA5‐85764, Invitrogen), NeuN (mouse, 1:100; #94403 S, CST), PSD95 (mouse, 1:100; #36233, CST), GAPDH (rabbit, 1:1000; ab181602, Abcam), Ki67 (rabbit, 1:1000; #9129, CST), FOXG1 (rabbit, 1:1000; ab3394, Abcam), Sox2 (rabbit, 1:200; #23064, CST), Pax6 (rabbit, 1:1000; #60433, CST), PAX6 (mouse, 1:200; ab78545, Abcam), OTX2 (rabbit, 1:200; #11943, CST), β3‐tubulin (mouse, 1:200; #4466, CST), MAP2 (rabbit, 1:200; #4542, CST), MAP2 (mouse, 1:200; ab11267, Abcam), and TBR1 (rabbit, 1:200; #49661, CST).

The following secondary antibodies were used: donkey anti‐rabbit immunoglobulin G (IgG) heavy‐chain and light‐chain (H&L) Alexa Fluor 488 (1:1000; ab150073, Abcam), donkey anti‐rabbit IgG H&L Alexa Fluor 488 (1:1000; ab150129, Abcam), donkey anti‐mouse IgG H&L Alexa Fluor 594 (1:1000; ab150108, Abcam), anti‐rabbit IgG (H&L), F(ab’)_2_ fragment Alexa Fluor 594 Conjugate (1:1000; #8890, CST), and goat anti‐rabbit IgG H&L Alexa Fluor® 488 (1:1000; ab150077, Abcam).

### 2.6 Western blotting

Total protein was isolated with radioimmunoprecipitation assay (Sigma–Aldrich) buffer supplemented with Protease Inhibitor Cocktail (Roche). Lysates were separated from the supernatant by centrifugation. Proteins were separated using SDS–polyacrylamide gel electrophoresis (PAGE) and transferred to a nitrocellulose membrane. For native PAGE, proteins were extracted with Nondenaturing Lysis Buffer (Sangon Biotech, C510013) and separated by native PAGE on a precast GLgel with HEPES Native‐PAGE (BBI, C601100). The samples were successively combined with primary antibodies and secondary antibodies conjugated with HRP. The secondary antibodies were PSD95 (rabbit, 1:1000; #3409, CST), anti‐rabbit IgG (H + L) (1:5000; #14708, CST), and goat anti‐rabbit IgG (1:5000; A0208, Beyotime).

### 2.7 Stereotactic injection

The microsyringe (2.5 μL) was rinsed 3–5 times with PBS. One microliter of air was aspirated into the microsyringe first to fully inject 0.25 μL of diluted virus into the brain. The syringe needle was placed according to the location parameter (Figure S1). The injection volume of ibotenic acid was 0.25 μL (10 μg/μL in saline), and the injection volume of viruses was 200 nL (30 nL/min). The Sham group was injected with the same amount of saline. The needle remained in place for an additional 8–10 min after injection.

### 2.8 Optogenetics

The viruses used in this experiment were AAV‐CaMKIIα‐ChR2‐mCherry and AAV‐CaMKIIα‐NpHR‐EGFP, provided by Hoyuan Biotechnology (Shanghai) Co. Optogenetic and electrophysiological experiments were performed 4 weeks later (Figure S2). The laser used in this experiment was yellow light of 590 nm wavelength and blue light of 470 nm wavelength. The laser power was 20 mW/mm^2^. Five‐minute stimulation periods were separated by 15‐min intervals. No laser stimulation was performed in the control group.

### 2.9 Chemogenetics

The virus used in this experiment was AAV‐CaMKIIα‐hM4D‐mCherry with a titer of 2.34 × 10^13^ vg/mL, provided by Hoyuan Biotechnology (Shanghai) Co. Clozapine‐N‐oxide (CNO, 1 mM) was injected intraperitoneally at 1 mg/kg 4 weeks after virus injection, and behavioral experiments were performed 45 min later. Sham group animals were injected with saline.

### 2.10 EEG–EMG monitoring and analysis

The EEG recording electrodes were implanted after anesthesia, and a reference electrode was placed on the forehead. The EMG recording electrodes were implanted in the neck muscles. EEG–EMG recordings were performed with the recording system (Pinnacle), and the data were analyzed with EEGLAB (v2019.1). The EEG–EMG signals were amplified (Grass Link, Grass Technologies), filtered (EEG: 0.3–30 Hz, EMG: 20–200 Hz), and digitized at 128 Hz using sleep recording software (Vital Recorder, Kissei Comtec). The polysomnography signals were automatically analyzed by analysis software (SleepSign for Animals, Kissei Comtec) and EEGLAB. The scoring results were visually inspected and manually corrected if necessary. The LOC and awake‐state classifications were performed by a researcher who did not participate in the experimental manipulation (Figure S2).

### 2.11 Behavioral tests

#### 2.11.1 Novel object recognition test

The experimental procedure was performed as described previously.^23^ Three objects were used. Objects A and B were identical, while object C was completely different from the others. Objects A and B were placed in symmetrical left and right positions in the field to begin the training. The recording software was turned on once the animal entered the room, and the recording time was 5 min. Object B was replaced with object C in a later test. The animal was put into the test room as above and recorded for 5 min to observe its exploration of object C. The exploratory preference was calculated as the percentage of time spent investigating the novel object in the total time spent exploring objects.

#### 2.11.2 Y‐maze test

The experimental procedure was performed as described previously.^24^ The Y‐maze apparatus consists of three identical arms. The animal was placed on the endpoint of a random arm and allowed to explore freely for 10 min. A camera recorded the changes in the behavior of the animal, including the following indicators: the number of times entering the Y‐arm (entry was defined as all feet of the mouse entering the Y‐arm), the number of correct alternations (the animal entered all three arms once in succession), the maximum rotation times (times of arm entries minus 2), and the correct reaction rate (number of correct alternations/maximum number of rotations × 100%).

#### 2.11.3 Adhesive removal test

The experimental procedure was performed as described previously.^25^ Two sticky papers were applied to the paws of the mice. The mouse was gently placed in the test box, and two timers were started. The time when the mouse began shaking or licking its paw was recorded as the start time, and the time when the mouse removed the sticky paper was recorded as the stop time. Bilateral paws were recorded, and the total time required to remove the sticky papers was calculated.

### 2.12 Statistical analysis

The data are presented as the mean ± SEM. Cell counting was performed using ImageJ (NIH). All data were analyzed with the independent‐sample *t* test in Prism 6 (GraphPad). The data were analyzed for normality using the D'Agostino–Pearson test, and for data that did not conform to a normal distribution, we used the Kruskal–Wallis test. *p < 0.05, **p < 0.01, ***p < 0.001, and ****p < 0.0001 were considered to be statistically significant.

## 3 RESULTS

### 3.1 A possible paradigm for a mouse model of DOC

Disorders of consciousness (DOC) are a common complication following traumatic brain injury (TBI) worldwide; however, there is a lack of rodent models. We attempted to generate a mouse model of DOC on the basis of controlled cortical injury (CCI), as we described in Section 2 (Figure 1A,B). In the following text, we refer to this model as CCI‐induced DOC (CCI‐DOC).

#### 3.1.1 Consciousness score decreases with increasing traumatic impact depth

For more visual statistics of the degree of DOC, we assigned each level of consciousness a corresponding score for statistical purposes, and levels I, II, III, IV, V, and VI were scored 6, 5, 4, 3, 2, and 1, respectively (Figure 1C). We found that the consciousness score of the CCI‐DOC mice decreased with increasing impact depth (Figure 1D). The consciousness score of mice decreased sharply once the impact depth exceeded 2.5 mm (2.5 mm group vs. Sham group, *p* < 0.001; 3 mm group vs. Sham group, *p* < 0.001; and 3.5 mm group vs. Sham group, *p* < 0.001). All of the consciousness scores in the 3, 3.5, and 4 mm groups were lower than 2 points.

#### 3.1.2 The survival rate of CCI‐DOC mice decreases with increasing impact depth

To better ensure the successful establishment of our model, we also determined the survival rate of mice. We found that when the impact depth exceeded 2 mm, the survival rate of CCI‐DOC mice decreased as the impact depth increased. The mortality rate of CCI‐DOC mice increased sharply when the impact depth exceeded 3 mm, and the 14‐day survival rate was significantly lower than 50% (Figure 1E). Therefore, in combination with the state of consciousness score (Figure 1D), we chose an impact depth of 3 mm, an impact speed of 3 m/s, and a duration of action of 180 ms as the modeling criteria for this experiment. This paradigm was used in all subsequent experiments.

#### 3.1.3 Prolonged awakening latency and increased duration of LOC

To test the effect of CCI‐DOC on awakening ability, we performed EEG–EMG recordings on CCI‐DOC mice and analyzed the statistics with EEGLAB software. The results showed the following: an animal's neural reflex (righting reflex) combined with EEG–EMG data could accurately determine the duration of loss of consciousness (LOC) and the latency to wake in the experimental animals (Figure 1F); the duration of LOC in the experimental animals gradually increased with increasing cortical impact depth (Figure 1G); and there was a statistically significant difference between the CCI‐DOC group and the Sham group (3 mm group vs. Sham group, *p* < 0.001; 3.5 mm group vs. Sham group, *p* < 0.001; 4 mm group vs. Sham group, *p* < 0.001).

#### 3.1.4 Cognitive decline, memory loss, and loss of limb sensation

To test the effect of CCI‐DOC on the content of consciousness, we performed a series of behavioral experiments. After 5 days of CCI‐DOC, we performed the novel object recognition experiment, Y‐maze experiment, and adhesive removal test. The results showed that the discrimination index of the CCI‐DOC group was significantly decreased compared with that of the Sham group (*p* < 0.001; Figure 1H). In addition, animals in the CCI‐DOC group showed a reduced frequency of exploration (Figure 1H,K). The results of the Y‐maze experiment showed that memory was impaired and the correct alternating reaction rate was decreased in the CCI‐DOC mice (CCI‐DOC group vs. Sham group, *p* < 0.001; Figure 1I). The adhesive removal performance showed that limb sensation was significantly reduced in CCI‐DOC mice, and the time to removal was significantly prolonged (CCI‐DOC group vs. Sham group, *p* < 0.001; Figure 1J).

### 3.2 A series of changes after CCI‐DOC

#### 3.2.1 Neuronal apoptosis in the PVT and CLA

**FIGURE 2:**
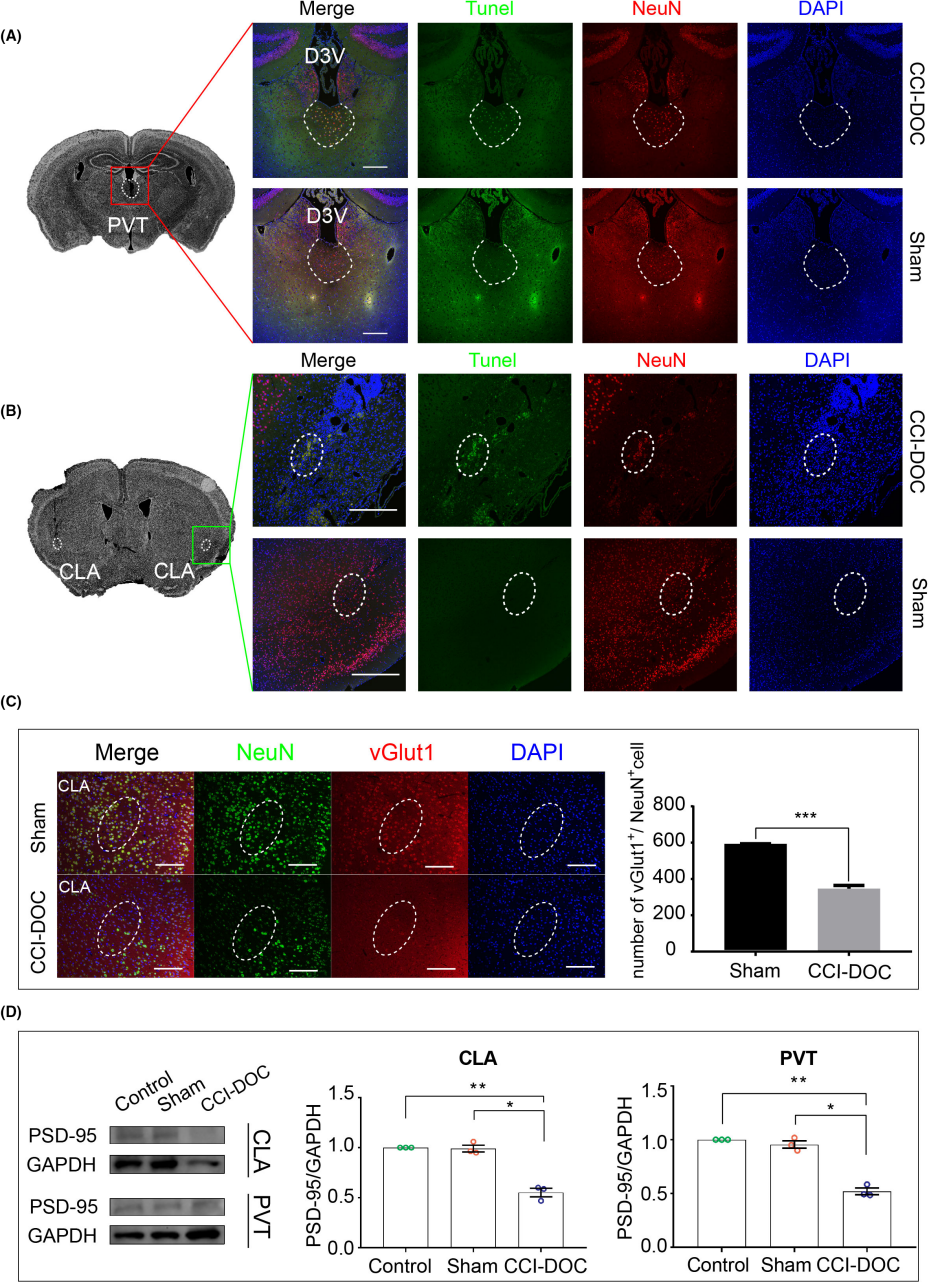
Damage to glutamatergic neurons associated with CCI‐DOC. (A) TUNEL/NeuN immunofluorescence staining in the PVT, bar = 200 μm. White virtual coils are the PVT. D3V: third ventricle. (B) TUNEL/NeuN immunofluorescence staining in the CLA, bar = 200 μm. White virtual coils are the CLA. (C) vGlut1/NeuN immunofluorescent double‐label staining revealed that the reduced neurons were mainly glutamatergic neurons. Significant reduction in glutamatergic neurons. Error bars: SEM. Significance was determined by Student's *t* test: ***p < 0.001, n.s., nonsignificant. *p* > 0.05. (D) Western blot results of PSD‐95 expression in CLA and PVT areas (*n* = 3 for each group).

Glutamatergic neurons have been found to be the predominant neuron type within the CLA^26^, ^27^ and PVT.^28^ TUNEL/NeuN immunofluorescent double‐label staining was used to detect apoptosis, and neuronal apoptosis was found in the PVT (Figure 2A) and the CLA (Figure 2B) in the CCI‐DOC mice. We questioned whether the DOC of mice was associated with the loss of glutamatergic neurons. We found with vGlut1/NeuN immunofluorescent double‐label staining that the reduced neurons in the CLA were mainly glutamatergic neurons (Figure 2C), and we further speculated that the activity or function of glutamatergic neurons might be associated with DOC. To further evaluate the role of glutamatergic neurons in consciousness, we detected postsynaptic dense protein PSD‐95, which is an important scaffold protein of glutamatergic neurons. The results showed that the expression level of PSD‐95 protein significantly decreased in both CLA slices and PVT slices of the CCI‐DOC mice (Figure 2D). Thus, we speculated that the activity or function of glutamatergic neurons might be associated with impaired consciousness. Therefore, we performed the same assay on other brain regions except for the traumatic brain impact area, where PVT and CLA neurons had the most apoptosis and other brain regions had a nonsignificant increase in neuronal apoptosis. Next, we also used optogenetic techniques to further reveal the role of glutamatergic neurons in DOC (Figure 4), before which we first had to clarify how the two nuclei function in DOC.

### 3.3 Arousal capacity and content of consciousness were also altered after stereotactic ibotenic acid destruction of the PVT and CLA

To further verify the relationship between these two nuclei and DOC, we performed neurotoxin destruction experiments on each of these two nuclei (PVT and CLA).

#### 3.3.1 Assessment results in awakening ability

**FIGURE 3:**
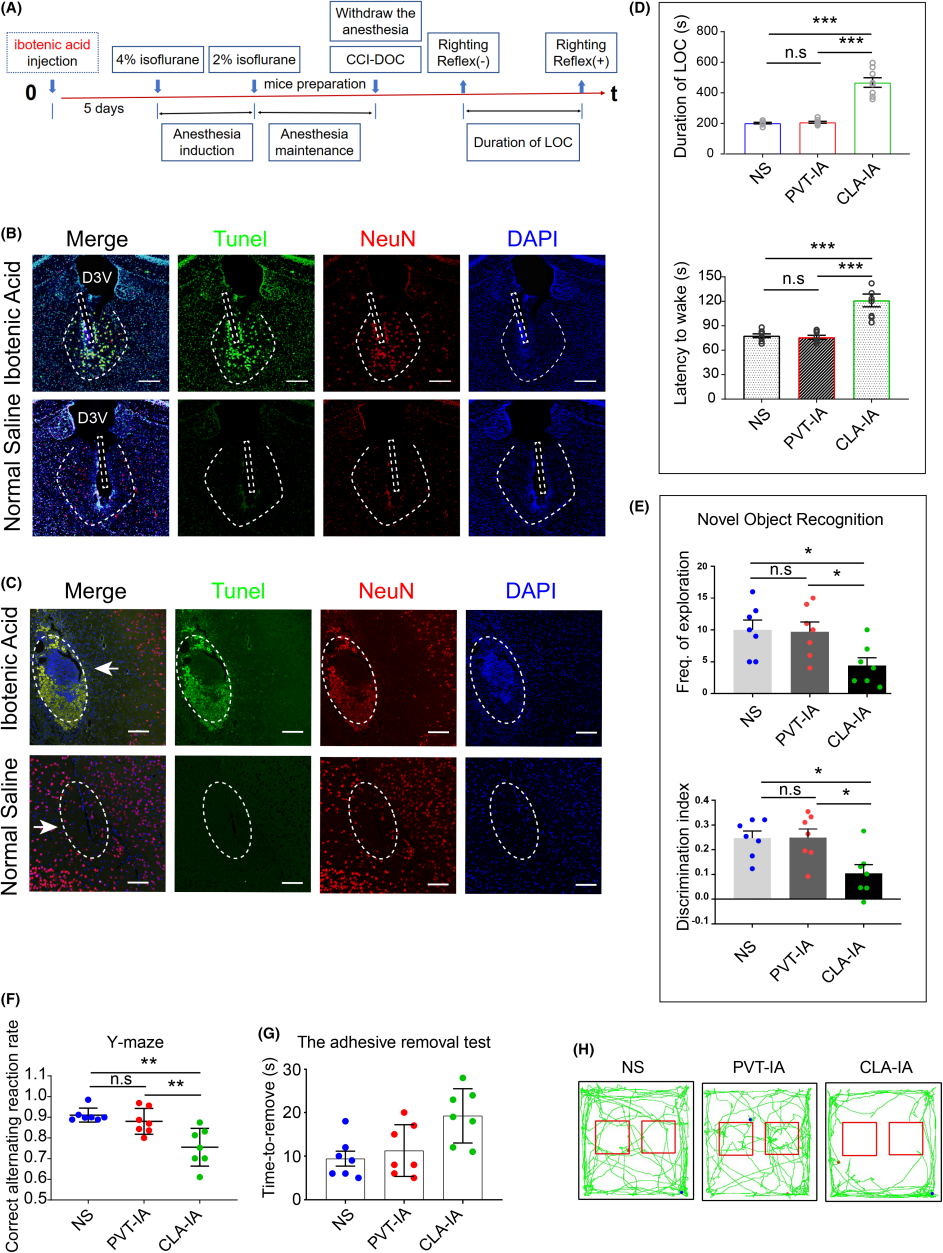
Paraventricular thalamus (PVT) glutamatergic neuronal damage leads to a significant decrease in wakefulness, and CLA glutamatergic neuronal damage leads to cognitive decline, memory loss, and limb sensory impairment. (A) Flow chart of this part of the experiment. (B) TUNEL/NeuN immunofluorescence staining of the PVT. The white rectangle indicates the needle tract bar = 200 μm, D3V: third ventricle. (C) TUNEL/NeuN immunofluorescence staining in the CLA. White arrows indicate needle tracts, bar = 100 μm. (I) Duration of LOC after neurotoxin disruption. (D–H) Assessment of consciousness after neurotoxic damage (*n* = 7 for each group). Error bars: SEM. Significance determined by Student's *t* test: ***p < 0.001, n.s. *p* > 0.05. The impact depth was 3 mm, the impact speed was 3 m/s, and the duration of action was 180 ms in (B–H).

Five days after stereotactic injection of ibotenic acid, the arousal capacity and consciousness of mice were impaired (Figure 3A). After execution, their brain tissues were double labeled by TUNEL/NeuN immunofluorescence. The results showed that a large number of apoptotic neurons were severely damaged in the PVT (Figure 3B) and CLA (Figure 3C). The duration of LOC and the latency to wake were not significantly different (CLA‐IA group vs. NS group, *p* > 0.05; Figure 3D). However, the PVT‐IA group showed a statistically significant difference in the duration of LOC and the latency to wake (PVT‐IA group vs. NS group, *p* < 0.05; Figure 3D).

#### 3.3.2 Assessment results in conscious content

The results of the novel object recognition experiment revealed that both the frequency of exploration and the discrimination index of the mice decreased significantly after the destruction of the CLA (CLA‐IA group vs. NS group, *p* < 0.05; Figure 3E,H), but there was no significant decrease in the PVT‐IA group (vs. NS group, *p* > 0.05; Figure 3E,H). The results of the Y‐maze experiment revealed that the memory of the mice was significantly reduced after the destruction of the CLA (CLA‐IA group vs. NS group, *p* < 0.05; Figure 3F), and the correct alternating reaction rate of the PVT‐IA group was not significantly different (vs. NS group, *p* > 0.05; Figure 3F). The results of the adhesive removal test showed that the time to removal was significantly prolonged in the CLA‐IA group (vs. NS group, *p* < 0.05; Figure 3G). The limb sensation of the mice in the PVT‐IA group was not significantly different (vs. NS group, *p* > 0.05; Figure 3G).

### 3.4 Inhibition of glutamatergic neurons aggravated DOC

#### 3.4.1 Optogenetic experiments

**FIGURE 4:**
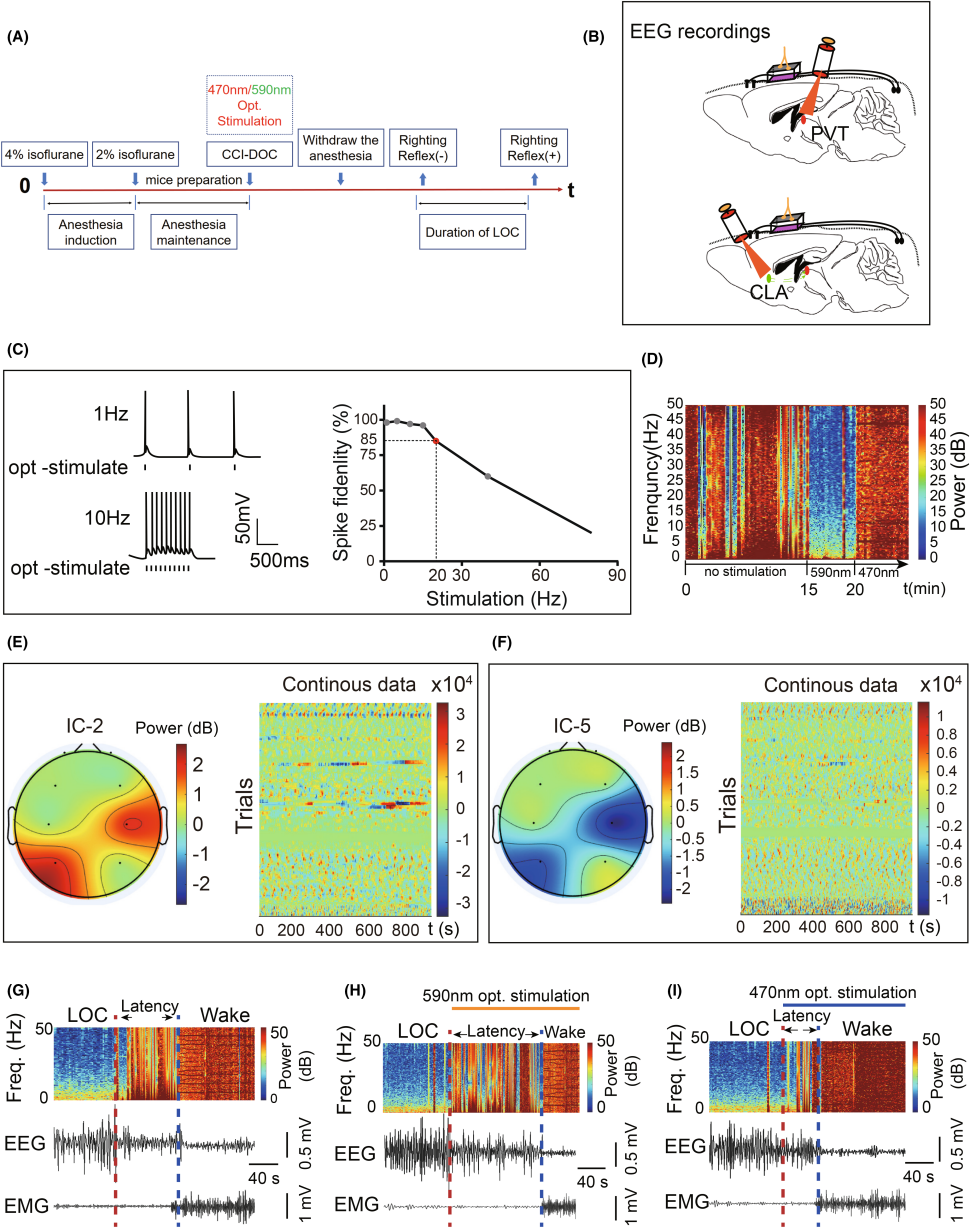
Activation of glutamatergic neurons induces arousal from DOC. (A) Flow chart of this part of the experiment. (B) Schematic diagram of the optogenetics experiment. (C) Example traces (left) and the fidelity of action potential firing (right) of ChR2‐expressing neurons evoked by 470 nm light stimulation with different frequencies. ChR2, channelrhodopsin‐2. (D) Glutamatergic neurons exhibit synchronized firing frequencies in response to optogenetic stimulation. (E, F) EEG energy and EEG–EMG recordings in controls after CCI‐DOC under 10 Hz light frequency stimulation 470 nm optogenetic activation of glutamatergic neurons and 590 nm inhibition of their activity. (G–I) EEG–EMG recordings under optogenetic stimulation. The duration of LOC was significantly reduced under 470 nm light stimulation, and the latency to wake was also significantly shortened.

To determine the role of glutamatergic neurons in the regulation of consciousness, we performed an optogenetic experiment (Figure 4A,B). Cells in the corresponding brain regions showed firing responses consistent with the stimulation frequency after stimulation with different frequencies of light (Figure 4C). When the light stimulation frequency was greater than 20 Hz, the fidelity of action potential firing appeared to decrease significantly as the stimulation frequency increased (Figure 4C); therefore, a 10 Hz light stimulation frequency was chosen in this experiment to ensure the fidelity of cell firing and a smaller possibility of damage (Figure 4C). The EEG of freely moving mice in the awake state in the normal group had stable basal waves and a neat rhythm. The EEG of the mice changed significantly after laser stimulation (Figure 4D). The frequency of EEG decreased and the power decreased after the administration of 590 nm yellow light stimulation (Figure 4D–F); and the frequency of EEG increased and the power increased after the administration of 470 nm blue light stimulation (Figure 4D–F). Thus, different wavelengths of laser light can be used to control the function of glutamatergic neurons.

#### 3.4.2 Assessment results in awakening

**FIGURE 5:**
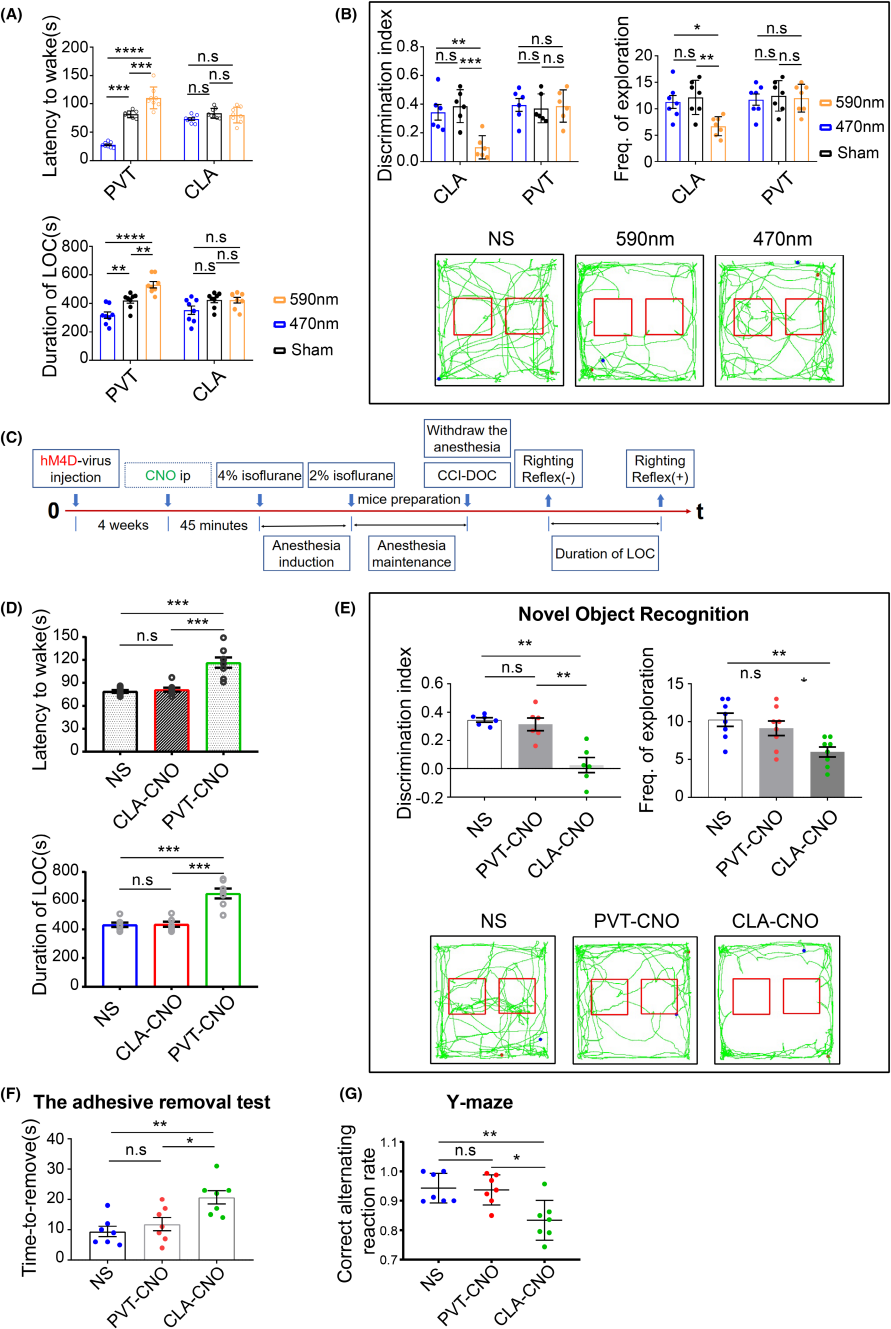
Paraventricular thalamus (PVT) glutamatergic neurons play an important role in arousal, and CLA glutamatergic neurons are important in the production of conscious content. (A) The duration of LOC and the latency to wake decreased under 470 nm light stimulation in the PVT (*n* = 8 for each group). The inhibition of excitatory neurons by 590 nm light in the PVT resulted in a statistically significant increase in the duration of LOC and the latency to wake (*n* = 8 for each group). (B) Under 590 nm light inhibiting excitatory neurons in the CLA, the discrimination index and the frequency of exploration decreased significantly (*n* = 6 for each group). (C) Flow chart of chemogenetics experiments. (D) After CNO inhibition of excitatory neurons in the PVT, a significant decrease in the latency to wake was shown as the duration of LOC was increased (*n* = 7 for each group). (E) After CNO inhibition of excitatory neurons in the CLA, a significant decrease in the discrimination index was shown (*n* = 6 for each group). (F, G) Memory loss after CNO inhibition of excitatory neurons; decreased limb sensation after CNO inhibition of excitatory neurons (*n* = 7 for each group). Error bars: SEM. Significance determined by Student's *t* test: ***p < 0.001, n.s. *p* > 0.05. NS, normal saline.

The duration of LOC increased after 590 nm light inhibition of glutamatergic neurons in the PVT (vs. 470 nm group, *p* < 0.001; vs. Sham group, *p* < 0.001; Figure 5A), and the latency to wake was prolonged, but there was no statistically significant difference in the CLA (vs. 470 nm group, *p* > 0.05; vs. Sham group, *p* > 0.05; Figure 5A). The duration of LOC was significantly reduced after the activation of glutamatergic neurons in the PVT by 470 nm light (vs. 590 nm group, *p* < 0.001; vs. Sham group, *p* < 0.001), and the latency to wake was prolonged, but there was no significant difference in the CLA (vs. 470 nm group, *p* > 0.05; vs. Sham group, *p* > 0.05; Figure 5A).

#### 3.4.3 Chemogenetics experiments

Clozapine‐N‐oxide inhibition of the PVT was followed by an increased duration of LOC and prolonged awakening latency.

We performed follow‐up experiments 4 weeks after hM4D virus injection in the PVT or CLA; 45 min prior to each experiment, mice were injected intraperitoneally with CNO (Figure 5C). We found a significant increase in the duration of LOC in mice after the inhibition of the PVT, and we found similar results for the latency to wake (vs. NS group, *p* < 0.001; vs. CLA‐CNO group, *p* < 0.001; Figure 5D). The duration of LOC was not significantly altered after CLA inhibition, and the latency to wake was not significantly altered (vs. NS group, *p* > 0.05; Figure 5D).

#### 3.4.4 Cognitive decline, memory loss, and diminished limb sensation after CNO inhibition of the CLA

After the inhibition of glutamatergic neurons within the CLA by CNO, the mice showed a significant decrease in the discrimination index and the frequency of exploration (vs. NS group, *p* < 0.01; vs. PVT‐CNO group, *p* < 0.05; Figure 5E), but no statistically significant difference was observed after the PVT was inhibited (vs. NS group, *p* > 0.05; Figure 5E). In addition, in the adhesive removal test and Y‐maze test, the mice in the CLA‐CNO group performed even worse after the inhibition of glutamatergic neurons within the CLA by CNO (vs. NS group, *p* < 0.01; vs. PVT‐CNO group, *p* < 0.05; Figure 5F,G).

### 3.5 Acquisition of glutamatergic neuronal/precursor cells through the induction of iPSCs *in vitro*

**FIGURE 6:**
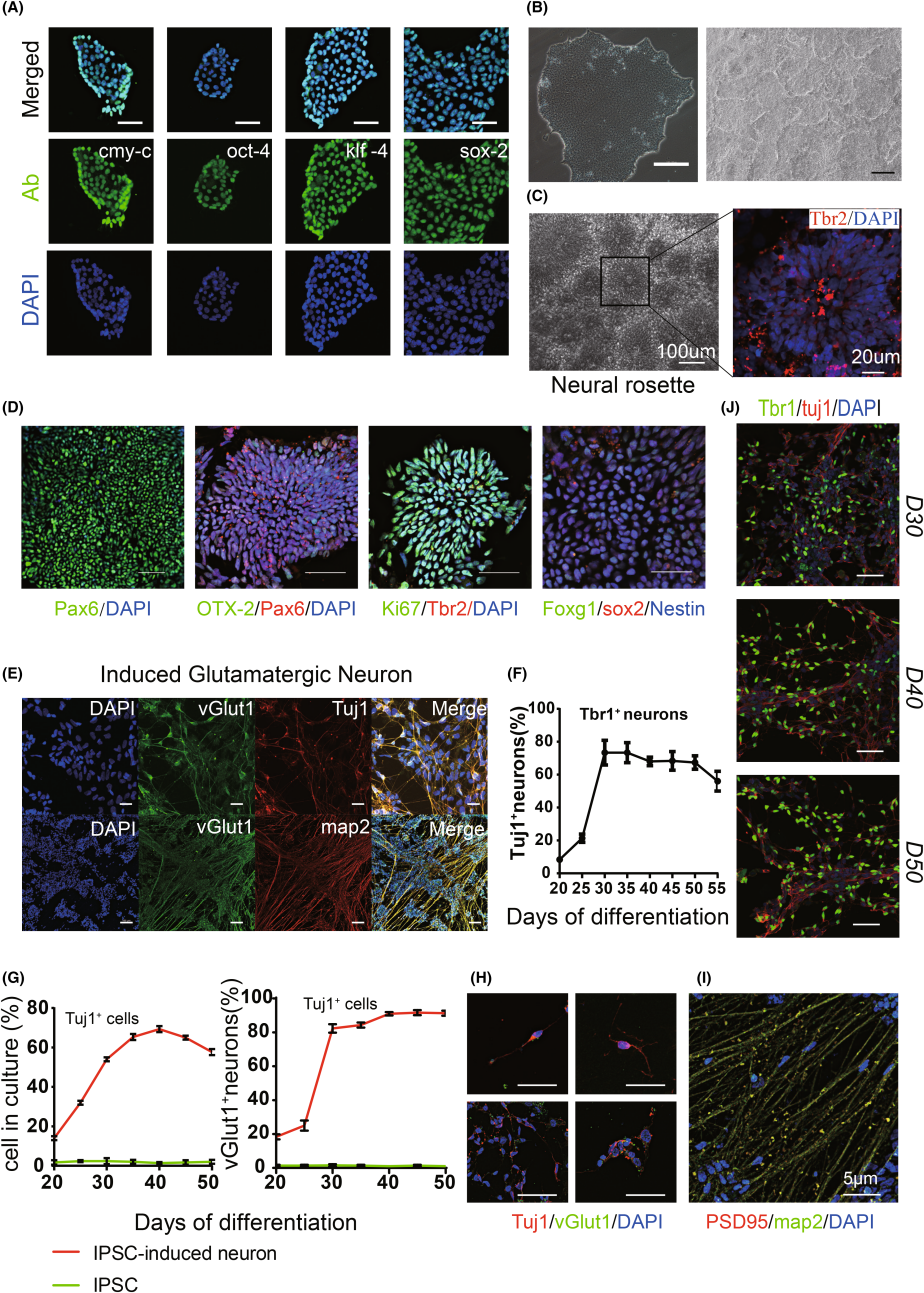
Acquisition of glutamatergic neuronal precursor cells by induced differentiation of iPSCs *in vitro*. (A) The four factors (cmy‐c/oct‐4/klf‐4/sox‐2) were transplanted into human fibroblast cells, bar = 50 μm. (B) (Left) iPSCs in good growth conditions and (Right) the formation of the neuroepithelial layer. (C) Typical neural rosette structure. (D) Immunofluorescence staining to confirm the cortical identity of induced neural tissue (Pax6, OTX‐2, Ki67, Tbr2, sox2, and nestin) is shown. (E) Confirmation of the glutamatergic identity of neurons by immunostaining for vesicular glutamate transporter 1 (vGlut 1, green) and neuron‐specific tubulin (Tuj1, red). (F) Glutamatergic neuronal precursor cells began to appear approximately 30 days after induction and began to decrease after Day 50. (G) The vast majority of cells obtained by induction were neurons (Tuj1+), and the proportion of these glutamatergic neurons (vGlut1+/Tuj1+) peaked at approximately Day 40 after induction. (H, I) Confirmation of mature glutamatergic neurons by immunofluorescent staining for neuronal markers (Tuj1, Glut1, PSD95, and map2). (J) Confirmation of glutamatergic neuronal precursor cells by immunofluorescent staining for specific markers (Tbr1 and tuj1). Nuclei are stained with DAPI. Scale bar, 50 μm.

The results of immunofluorescence staining showed that the cells induced from iPSCs (Figure 6A,B) *in vitro* showed cortical neuron–specific markers (Pax6^+^/Ki67^+^/OTX2^+^/foxg1^+^/sox2^+^/Tbr2^+^) (Figure 5C,D), and the glutamatergic neuron‐specific marker vGlut1 staining was positive as well; most of the cells induced by this method were neurons (Tuj1^+^) (Figure 6E,F), and the proportion of glutamatergic neurons (vGlut1^+^/Tuj1^+^) peaked at more than 90% on the 40th day after induction (Figure 6G). The proportion of glutamatergic neuronal precursor cells (Tbr1^+^/Tuj1^+^) was very high on Days 30–50 after induction (Figure 6J). Glutamatergic neurons began to fully mature (map2^+^/PSD‐95^+^) approximately 50 days after induction (Figure 6H,I).

### 3.6 Acquired glutamatergic neurons exhibit excitatory neuronal electrophysiological properties

**FIGURE 7:**
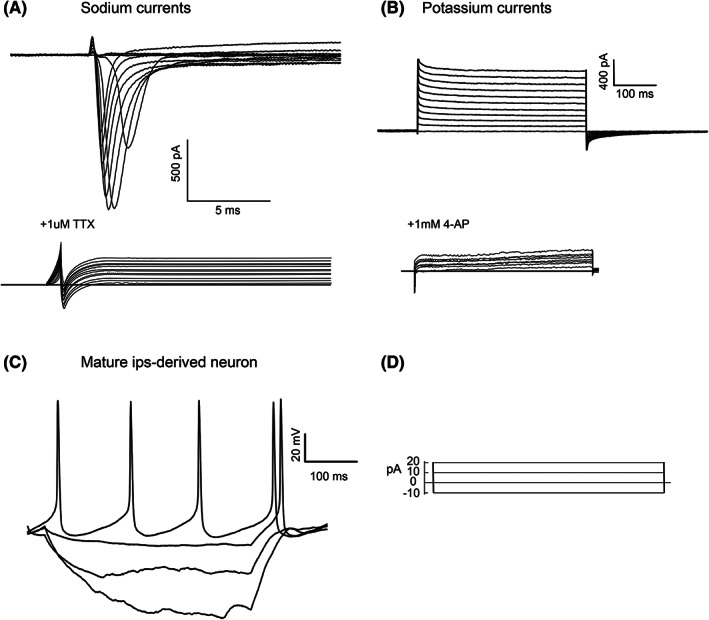
Whole‐cell patch clamp showed that induced neurons have excitatory neuronal electrophysiological properties. (A, B) Mature sodium and potassium channels. Whole‐cell patch clamp recordings of individual cells showed that in voltage‐clamp mode, sodium and potassium currents were examined by performing a series of step depolarizations from a holding potential of −80 to +40 mV, voltage‐gated sodium channels could be blocked by 1 μM tetrodotoxin (TTX), and voltage‐gated potassium channels could be blocked by 1 mM 4‐AP. (C, D) By performing stepwise current injection from −20 to +60 pA and examining the action potential in current‐clamp mode, the results showed that our induced glutamatergic neurons had mature excitable neuronal action potentials.

Whole‐cell patch clamp results showed that normal Na^+^ currents and K^+^ currents were successfully recorded in cortical neurons induced from iPSCs *in vitro* (Figure 7A,B); Na^+^ currents could be blocked by TTX and K currents could be blocked by 4‐AP (Figure 7A,B). In addition, cortical neurons induced from iPSCs showed strong, regular action potentials in response to step current injection (Figure 7C,D).

### 3.7 Transplanted glutamatergic neuronal precursor cells survive, develop further in the brain, and function as excitatory neurons

**FIGURE 8:**
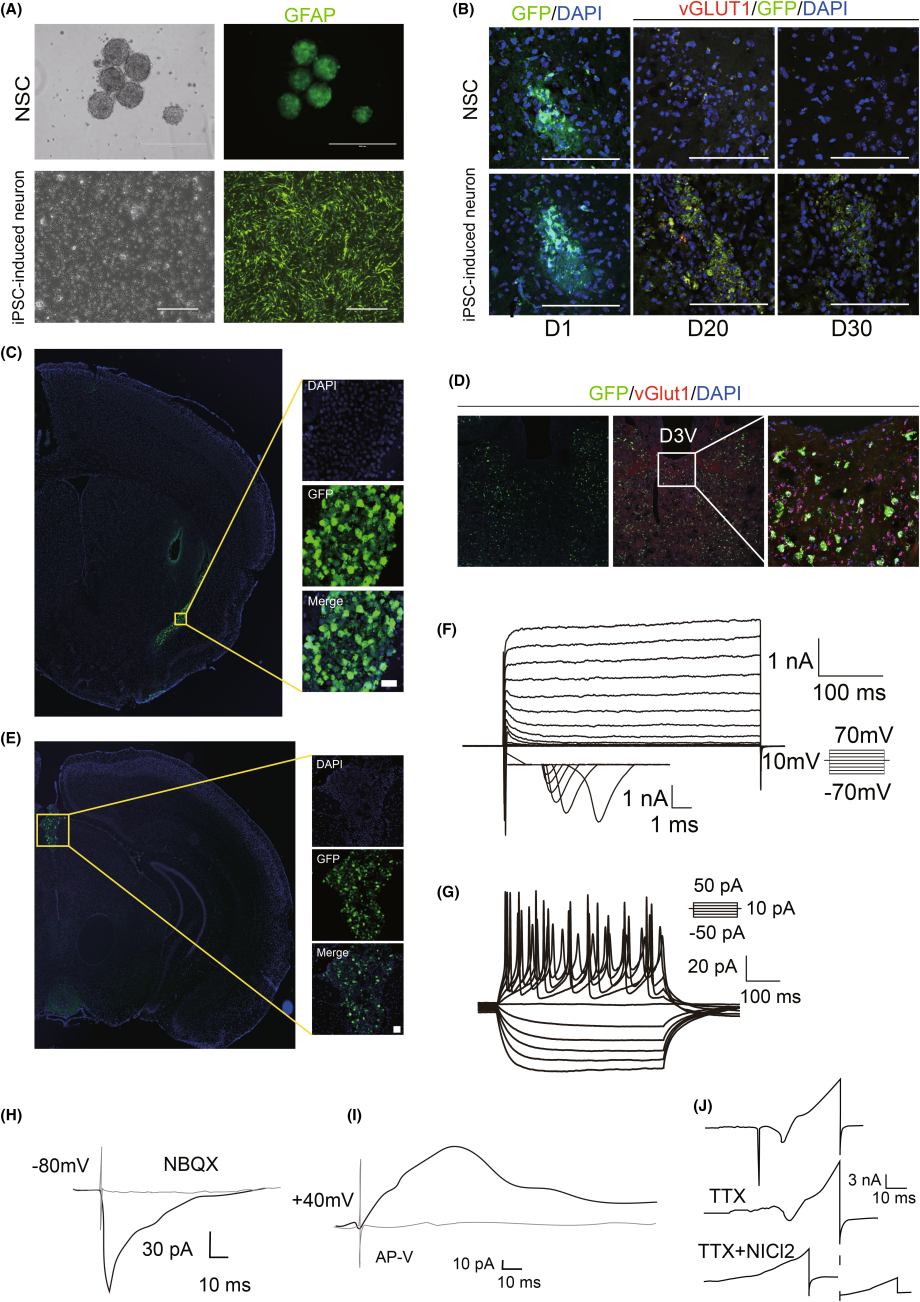
Glutamatergic neuronal precursor cells can survive and develop further into mature excitatory neurons after transplantation. (A) Green fluorescent labeling of NSCs and glutamatergic neuronal precursor cells. (B–E) Confirmation of the location of glutamatergic neuronal precursor cells in the CLA and PVT by immunofluorescence after transplantation. (F, G) Action potentials (APs) and Na‐ion currents can be recorded in the cell transplantation area. (H, I) Aminergic neuron–specific excitatory postsynaptic currents (EPSCs) disappear when APV/NBQX (NMDA and AMPA receptor recruiting agent) is added. (J) Excitatory neural precursor cells can be inhibited by (TTX + NiCl_2_).

First, we infected the acquired glutamatergic neuronal precursor cells with viruses carrying green fluorescent tags. Twenty‐four hours after GFP^+^ viral transfection, glutamatergic neurons emitted green fluorescence (GFP^+^) (Figure 8A). Second, we successfully transplanted glutamatergic neuronal precursor cells into the PVT and CLA. We performed immunofluorescence staining of brain tissues on Days 1, 20, and 30 after transplantation. A large number of green fluorescent (GFP^+^)‐labeled glutamatergic neurons were found in the CLA (Figure 8B,C) and PVT (Figure 8D,E) after the staining of brain tissue sections. This finding indicated that we successfully transplanted cells into the brain. We further verified our conclusion by electrophysiological experiments showing that iPSC‐derived neurons survived in the mouse brain and exhibited mature glutamatergic neuronal properties after transplantation. Thirty days after transplantation, we euthanized the animals, rapidly removed their brain tissue, and sectioned the tissue for brain slice patch‐clamp assays. The patch‐clamp results showed that normal Na^+^ and Ca^+^ currents and normal postsynaptic currents of excitatory neurons after transplantation were recorded after transplantation (Figure 8F–J). The excitatory postsynaptic current (EPSC) could be inhibited by NBQX/AP‐V (Figure 8H). These results indicate that transplanted glutamatergic neuronal precursor cells survive and further develop in the brain and function as excitatory neurons.

### 3.8 Transplanted glutamatergic neuronal precursor cells promote awakening and improve conscious content

**FIGURE 9:**
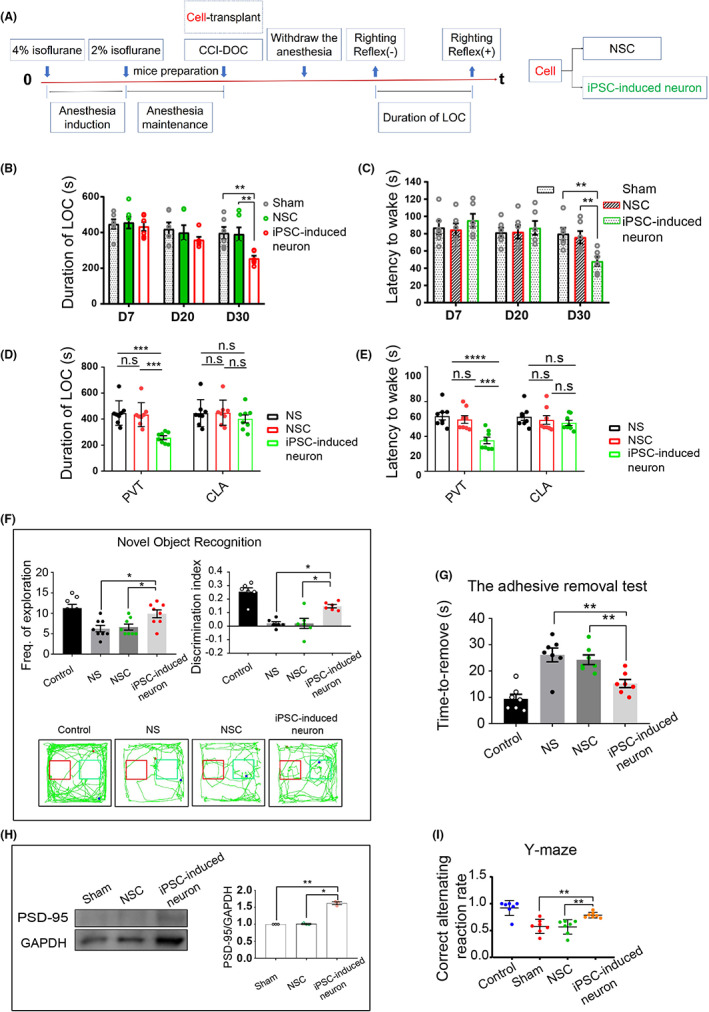
Glutamatergic neuronal precursor cell transplantation significantly improves wakefulness and facilitates recovery of conscious content. (A) Flow chart of the cell transplantation experiment. (B, C) At 7, 20, and 30 days after cell transplantation in the PVT, we performed the awaking ability assessments. The results showed that in the iPSC‐induced neuron group, the duration of LOC was statistically reduced, and the latency to wake was shortened (*n* = 6 for each group). (D, E) We compared the effects of transplantation into the CLA and PVT on awaking ability, and the results showed that in the iPSC‐induced neuron transplantation groups, the PVT group showed significantly enhanced arousal, while the CLA group did not show a statistically significant difference in awaking ability (*n* = 8 for each group). (F) After transplantation of iPSC‐induced neurons, the discrimination index improved significantly (*n* = 6 for each group), and the frequency of exploration was enhanced (*n* = 8 for each group). (G, I) In the CLA group, 30 days after iPSC‐induced neuron transplantation, the evaluation of conscious content was significantly improved (*n* = 7 for each group). (H) At 30 days after cell transplantation, elevated PSD95 expression was seen in the transplanted region, with significantly higher PSD95 expression in the iPSC‐derived neuron group. Error bars: SEM. Significance determined by Student's *t* test: ***p < 0.001, n.s. *p* > 0.05. LOC, loss of consciousness.

To better demonstrate the effect of glutamatergic neuronal precursor cell transplantation, we used neural stem cells (NSCs) as a control group. After generating the CCI‐DOC model, we randomly divided the animals into three groups: the Sham group, the transplanted NSC group, and the transplanted iPSC‐derived neuron group. Both the PVT and CLA groups underwent cell transplantation (NSC or iPSC‐derived neurons), with six mice in each group. At 7, 20, and 30 days after cell transplantation, isoflurane anesthesia induction experiments (anesthetic concentration 4%, duration 30 min) were performed (Figure 9A), and the duration of LOC and awakening latency were recorded. We first performed cell transplantation experiments on the PVT (Figure 9B,C), and we found that significant differences began to appear more than 30 days after cell transplantation. Therefore, subsequent experimental evaluations were performed 30 days after cell transplantation. In the iPSC‐induced neuron transplantation group, the awakening latency and the duration of LOC were shortened (vs. NS group, *p* < 0.01; vs. NSC group, *p* < 0.01; Figure 9B–E). In addition, we compared the effects of transplantation into the CLA and PVT on awaking ability, and the results showed that in the iPSC‐induced neuron transplantation groups, the PVT group showed significantly enhanced arousal, while the CLA group did not show a statistically significant difference in awaking ability (Figure 9D,E). In the CLA group, 30 days after iPSC‐induced neuron transplantation, the conscious content was significantly improved (vs. NS group, *p* < 0.01; vs. NSC group, *p* < 0.05; Figure 9F–H). In addition, the expression of PSD‐95 increased significantly (Figure 9I).

## 4 DISCUSSION

Disorders of consciousness (DOC) are a common and serious complication after severe traumatic brain injury (TBI). At present, there are few effective treatment methods for posttrauma DOC. Improving the prognosis and reducing the mortality of patients with chronic DOC after TBI is an urgent problem in modern society. Stem cell transplantation is a new direction in the treatment of DOC in recent years. However, the choice of transplantation target and cells is not clear. In the past, there have been many studies on disorders of consciousness, basically using anesthesia or natural sleep state models, which make it difficult to accurately simulate the real situation of DOC after TBI.

Disorders of consciousness (DOC) have always been a hotpot and difficulty in neuroscience research. DOC is recognized as an important part of the development and planning of brain research. Elucidating the mechanism of DOC is the top priority in solving the treatment problems of patients with DOC. According to classic neuroscience theory, the essence of consciousness includes two aspects: one is the state of consciousness, that is, the awakening ability, or the ability to maintain the basic awake state; the other is the content of consciousness, including cognitive ability, memory, sensory integration, and other advanced brain functions. Previous studies have suggested that the PVT plays an important role in the maintenance of wakefulness.^14^ Our study further revealed that the state of glutamatergic neurons in the PVT may determine its function and thus affect the state of consciousness (wakefulness); in addition, we also found that a large number of glutamatergic neurons are also present in the CLA, and these neurons are involved in the generation of conscious content and play an important role in the reconstruction and repair of synapses after DOC. When loss of consciousness occurs in mice, glutamine neurons in both nuclei undergo massive apoptosis.

Although an association between these two nuclei and consciousness has been reported,^14^, ^29^ to demonstrate the relationship between these two nuclei and the state and content of consciousness, we further demonstrated that these two nuclei control different aspects of consciousness by chemical and optogenetic means and that their destruction or activation can demonstrate the important role they play in their respective spheres of control. Since the CLA is a deep region of the brain, previous studies on CLA injury models are scarce. We used optogenetic techniques to precisely control the function of the CLA to achieve the inhibition of the CLA, which can achieve the effect of injury. The relationship of PVT and CLA with DOC has been less extensively demonstrated in animal models. Through this study, we found that the apoptosis of glutamatergic neurons in the PVT and CLA may be an important factor in the development of DOC, and better therapeutic effects were achieved by transplanting glutamatergic neuronal precursor cells, which suggests that promoting glutamatergic neuronal regeneration and repair is the key to the treatment of DOC.

Stem cell transplantation is a very promising therapeutic modality in the field of neuroprosthetics.^30^, ^31^, ^32^ However, previous stem cell transplantation methods, in which the stem cells themselves were directly transplanted into animals, would create a problem, which is the differentiation of stem cells. When cultured *in vitro*, the survival environment of stem cells is homogenous, controllable, and adjustable, which can ensure their normal differentiation; however, after transplantation into the body, the cellular microenvironment changes all the time, and the *in vivo* environment is more complex and unstable, which impacts the differentiation direction and efficiency of stem cells, and the uncertainty of differentiation direction increases greatly.^33^, ^34^ The current direction of research is precision transplantation, which means the differentiation of iPSCs into specific cell products *in vitro* and then transplanting the cell products, especially neural precursor cells, in patients with neurological diseases.^35^ Since glutamatergic neuroapoptosis occurs with DOC, the transplantation of glutamatergic precursor cells has the potential to play a compensatory role. We successfully induced iPSCs *in vitro*, further induced them to differentiate into glutamatergic precursor cells, and confirmed by electrophysiological and other methods that they have electrophysiological properties of neurons and can secrete glutamate. The transplantation of these precursor cells into the CLA and PVT can significantly improve DOC in comatose animals.

We propose that the mechanisms of DOC improvement may be as follows. First, transplantation directly replenished glutamatergic neurons, which released more excitatory neurotransmitters. Glutamate is the most abundant neurotransmitter in the vertebrate nervous system, and more than 90% of excitatory synapses in neurosynaptic connections use glutamate as a neurotransmitter for information transmission.^36^, ^37^

Furthermore, the transplanted glutamatergic neurons could form new synaptic connections with neurons within the neural network, which helped to repair key nodes that activated the conscious neural network. The findings of electrophysiological assays revealed that at approximately 30 days, the transplanted cells exhibited the electrophysiological properties of mature excitatory neurons: normal excitatory neuronal firing action potentials (APs) and normal Na and Ca currents could be detected in the transplanted area and could be blocked by the specific ion channel blocker TTX + NiCl_2_; excitatory postsynaptic currents were recorded in the transplanted area; and the addition of the NMDA/AMPA receptor blocking agents MAPV and MNBQX inhibited excitatory postsynaptic currents. These electrophysiological results demonstrate that transplanted glutamatergic neuronal precursor cells survive and further mature in the transplanted region. Multiple mature excitatory neurons could potentially interconnect and form synapses, which is consistent with our experimental results.

In addition, the PVT sends dense fiber projections to the NA (nucleus accumbens),^38^ which is involved in behaviors dependent on a heightened state of arousal, allowing excitability to continue to higher neural clusters, which are integrated by the CLA and eventually reach the neural correlates of consciousness (NCC) in the posterior midbrain cortex, which in turn activates cortical neurons and enhances cortical excitability and the ability to sense external stimuli, potentially facilitating arousal and recovery of conscious content in patients with DOC.

Our study has considerable value but also some limitations. We identified two different functions of the PVT and CLA in consciousness generation, but we did not further elucidate whether there is some connection between the two nuclei; we believe that there may be some potential circuits linking them, which will be the next topic for our team to explore in depth.

## 5 CONCLUSION

In summary, we first constructed a feasible CCI‐DOC model. We investigated the roles of PVT and CLA in the development of DOC. Our findings demonstrated that the transplantation of glutamatergic neuronal precursor cells into these sites promoted arousal and recovery of conscious content. This evidence implies that CLA and PVT are possible intervention targets for DOC. We thus offer a promising stem cell treatment for DOC that has great clinical research value.

## AUTHOR CONTRIBUTIONS

T.Z. designed the study. N.L.W. and T.Z. performed these experiments and analyzed the data. T.W.L. performed the immunofluorescence experiments. J.H.Z. conceived the study and provided valuable comments and reagents and edited the manuscript. K.Z.C., W.Q.C., Z.F.W., and F.W. bred the mice and performed the experiments. Y.X.L. provided valuable comments. T.Z., N.L.W., and T.W.L. analyzed the data and wrote the manuscript.

## CONFLICT OF INTEREST STATEMENT

The authors declare no competing interests.

## Supporting information


Figure S1.

Figure S2.

## Data Availability

The datasets generated during and/or analyzed during the current study are available from the corresponding author upon reasonable request.
