# Supplementary figures and images for: Transplantation of glutamatergic neuronal precursor cells in the paraventricular thalamus and claustrum facilitates awakening with recovery of consciousness
Source: CNS Neurosci Ther. 2023 Mar 7;29(7):1785–804. doi: 10.1111/cns.14137 (PMC10324366; doi:10.1111/cns.14137)

AP -1.22mm  
ML +/- 0.5mm  
DV -2.95mm  
with a 10° angle  
toward the midline

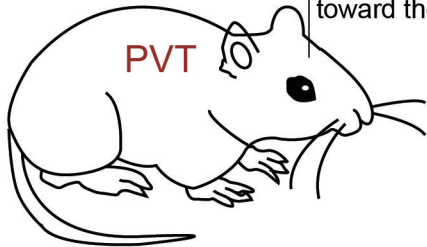

AP +1.1mm  
ML +/-2.5mm  
DV -3.75mm

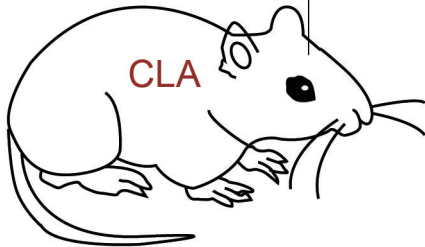

Supplement: Supplementary file 1 — Figure S1. [file CNS-29-1785-s001.pdf]

Acronym:PVT

Name:Paraventricular thalamus

500μm

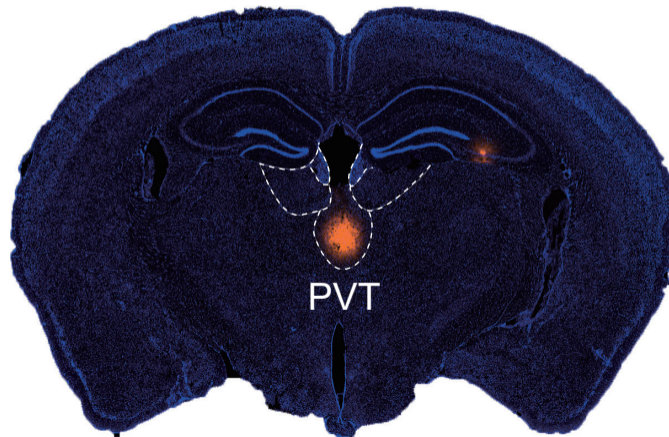

Acronym:CLA

Name:Claustrum

500μm

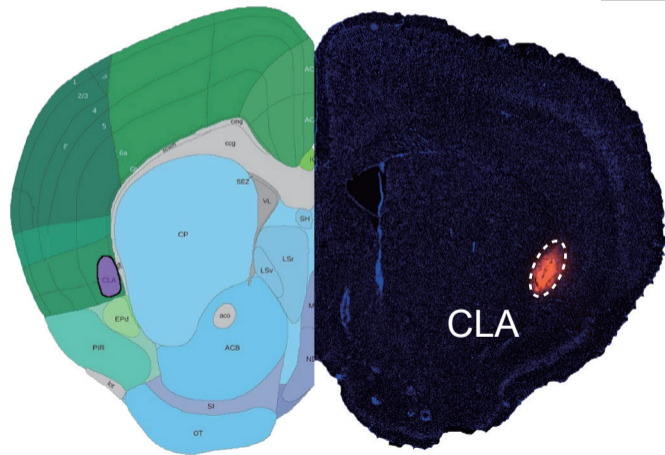

Supplement: Supplementary file 2 — Figure S2. [file CNS-29-1785-s002.pdf]
